# SERAPHIM 2.0: an extended toolbox for studying phylogenetically informed movements

**DOI:** 10.1093/bioinformatics/btag093

**Published:** 2026-02-24

**Authors:** Simon Dellicour, Nuno R Faria, Rebecca Rose, Philippe Lemey, Oliver G Pybus

**Affiliations:** Spatial Epidemiology Lab (SpELL), Université Libre de Bruxelles (ULB), Brussels 1050, Belgium; Department of Microbiology, Immunology and Transplantation, Rega Institute, KU Leuven, Leuven 3000, Belgium; Interuniversity Institute of Bioinformatics in Brussels, Université Libre de Bruxelles, Vrije Universiteit Brussel, Brussels 1050, Belgium; MRC Centre for Global Infectious Disease Analysis, Department of Infectious Disease Epidemiology, School of Public Health, Imperial College London, London W12 0BZ, United Kingdom; Institute of Tropical Medicine, University of São Paulo, São Paulo 05403-000, Brazil; BioInfoExperts LLC, Sid Martin Biotech, University of Florida, Alachua, FL 32615, United States; Department of Microbiology, Immunology and Transplantation, Rega Institute, KU Leuven, Leuven 3000, Belgium; Department of Biology, University of Oxford, Oxford OX1 3RB, United Kingdom; Department of Pathobiology and Population Science, Royal Veterinary College, Hatfield AL9 7TA, United Kingdom

## Abstract

**Summary:**

We report the second version of the R package “seraphim”, a toolbox developed to process and analyze the output of spatially explicit phylogeographic reconstructions. This approach – also known as continuous phylogeographic inference – is commonly used in molecular epidemiology to reconstruct the dispersal history and spatiotemporal dynamics of rapidly evolving pathogens. The “seraphim” package now implements a broad range of features including (i) visualization of phylogeographic inferences, (ii) estimation of lineage dispersal metrics, (iii) several phylogeographic simulators, and (iv) hypothesis testing procedures to investigate the impact of environmental factors on variables such as diffusion velocity, dispersal location, and dispersal frequency of phylogenetic lineages.

**Availability and implementation:**

The package is openly available (https://github.com/sdellicour/seraphim) along with a series of tutorials describing the different analytical procedures it implements.

## 1 Introduction

When placed in a spatio-temporal context, phylogenetic trees can constitute a valuable source of information about the dispersal history and dynamics of viruses, and is achieved through phylogeographic inference. Popular methods for phylogeographic inference are typically categorized into discrete versus continuous approaches (Baele *et al.* 2018). Discrete approaches – based on discrete trait analysis (Lemey *et al.* 2009) or structured coalescent approximations (De Maio *et al.* 2015, Müller *et al.* 2018, Müller *et al.* 2025) – are based on an a priori definition of a set of discrete sampling locations and usually requires the assumption that all ancestors of the sampled viruses existed only at locations belonging to that set (Dellicour *et al.* 2018b). While spatial discretization can be sometimes arbitrary and/or lead to over-simplification, discrete phylogeography approaches have however proven relevant and useful in a number of studies, e.g. when attempting to test hypotheses about the impact of external factors on the frequency of dispersal events among discrete locations through generalized linear modeling (Lemey *et al.* 2014, Dudas *et al.* 2017). Continuous phylogeographic methods (Lemey *et al.* 2010, Fisher *et al.* 2021, Guindon and De Maio 2021, Bastide *et al.* 2024) represent an alternative approach to the spatial reconstruction of virus spread. Continuous approaches are particularly relevant when the pattern of spatial virus dispersal maintains a relationship with geographic distance; this may not always be the case, e.g. when the global spread of human respiratory viruses is driven by international air traffic (Lemey *et al.* 2014). While discrete and continuous approaches represent complementary tools to reconstruct viral spread in space and in time, it is important to note that both approaches are impacted by sampling bias (Kalkauskas *et al.* 2021, Layan *et al.* 2023).

Continuous phylogeographic inference has been made popular by its implementation (Lemey *et al.* 2010, Pybus *et al.* 2012) in the software package BEAST for Bayesian phylogenetic inference (Drummond *et al.* 2012, Bouckaert *et al.* 2019, Baele *et al.* 2025). This continuous phylogeographic approach uses 2D relaxed random walk (RRW) diffusion models to infer the location – i.e. geographic coordinates – of ancestral nodes within phylogenetic trees, while allowing for branch-specific variation in dispersal velocity (Pybus *et al.* 2012, Dellicour *et al.* 2021). Continuous phylogeographic inference is widely used in molecular epidemiology to reconstruct the spread of fast-evolving pathogens, such as RNA viruses, and has been notably applied to a number of emerging infections of humans (Faria *et al.* 2018, Kraemer *et al.* 2021). Yet, while spatially explicit, these methods do not model nor attempt to identify the (continuous) environmental factors that may affect the dispersal process, which motivated the implementation of the first version of the “seraphim” toolbox (Dellicour *et al.* 2016a).

We here present a second, substantially updated version of our R package “seraphim”, which was developed to analyze phylogenetically informed movement inference through continuous phylogeographic analysis. Specifically, “seraphim” 2.0 can be used to (i) visualize continuous phylogeographic reconstructions, (ii) estimate lineage dispersal statistics, (iii) perform continuous phylogeographic simulations, and (iv) conduct various hypothesis tests on the impact of environmental factors on the dispersal dynamic of lineages. Our open-source R package is available on GitHub along with a series of tutorials and associated example files that describe and illustrate how to apply the different analytical procedures it provides.

## 2 Features

Since its first release ten years ago (Dellicour *et al.* 2016a), several new features and methods have been added to “seraphim” and these are described below (see also Table 1, available as supplementary data at *Bioinformatics* online for an overview of all the new and updated functions in the package). The first step of all the analytical frameworks implemented in “seraphim” remains the generation of spatio-temporal information by sampling phylogenetic trees from a posterior distribution of trees inferred through Bayesian continuous phylogeographic inference. After this step, each posterior tree is decomposed as an extraction table, in which each row corresponds to a distinct phylogenetic branch summarized as a movement vector (Pybus *et al.* 2012) with a duration, start and end times, as well as start and end locations. The resulting set of extraction tables then serve as the basis of all the subsequent visualizations and analyses that can be conducted with the toolbox.

### 2.1 Visualization of continuous phylogeographic reconstructions

**Figure 1:**
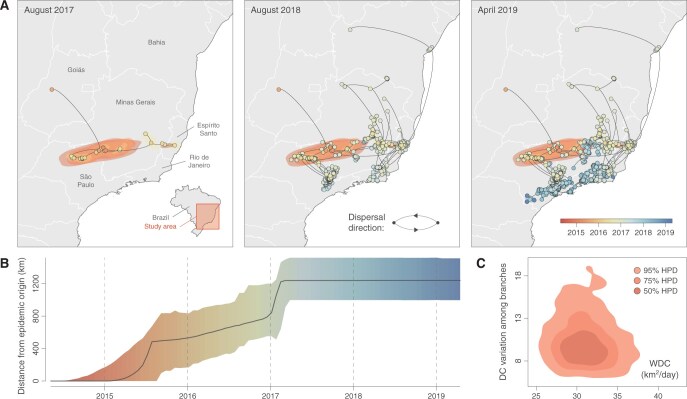
Examples of visualizations that can be generated with the toolbox “seraphim” 2.0. Visualizations are based on a continuous phylogeographic analysis of the yellow fever virus (YFV) outbreak that started around 2015 in southeastern Brazil (Hill *et al*. 2022). (A) Continuous phylogeographic reconstruction of the dispersal history of YFV outbreak lineages: maximum clade credibility (MCC) tree and overall 80% highest posterior density (HPD) regions reflecting the uncertainty of the Bayesian phylogeographic inference summarized from 1000 trees sampled from the post-burn-in posterior tree distribution. MCC tree nodes are colored according to their time of occurrence and 80% HPD regions were computed for successive time layers and then superimposed using the same color scale to reflect time. The underlying map delimiting the Brazilian states was retrieved from the Database of Global Administrative Areas (GADM; https://gadm.org). (B) Evolution of the maximal wavefront distance from the epidemic origin: the solid curve represents the median value and the surrounding polygon the 95% HPD interval. Those estimates are also based on 1000 trees sampled from the post-burn-in posterior tree distribution, and the uncertainty polygon is colored according to the same time scale used in panel A. (C) Evaluation of the diffusion velocity of viral lineages through the estimation of the weighted diffusion coefficient (WDC): kernel density estimates of the diffusion coefficient (DC) parameters, with the posterior WDC estimates on the *x*-axis and the coefficient of variation of the diffusion coefficient among the branches of each sampled tree on the *y*-axis. In this graph, the three contours show, in shades of decreasing darkness, the 50%, 75%, and 95% HPD regions via kernel density estimation, respectively.

The package “seraphim” offers a flexible framework to generate detailed and customizable visualizations of continuous phylogeographic reconstructions. The newly available “spreadGraphic1” and “spreadGraphic2” functions can be used to estimate highest posterior density (HPD) polygons that reflect the uncertainty associated with the Bayesian phylogeographic inference. While the “spreadGraphic1” function extracts the 80% HPD polygons associated with each internal node of an individual maximum clade credibility (MCC) tree (previously retrieved and annotated with the program TreeAnnotator; Baele *et al.* 2025), the “spreadGraphic2” function estimates HPD polygons corresponding to successive time slices while considering several posterior trees and all internal nodes falling in each time slice. Unlike the implementation available in the first version of “seraphim”, these uncertainty polygons can now be saved in a vectorial format (i.e. shapefiles) and reported alongside the mapping of the MCC tree to summarize a continuous phylogeographic reconstruction (see Fig. 1A for an example). The utility of a visualization conducted in R lies in the flexibility of the geographic and landscape features that users can choose to plot together with a phylogeographic reconstruction; such features might include administrative borders, water flows, and/or any environmental factors that may have impacted the spread of lineages (and which can be further investigated with the hypothesis testing approaches implemented in the toolbox; see below).

### 2.2 Estimation and comparison of dispersal statistics

The “spreadStatistics” function available in “seraphim” has been updated to allow the estimation of an extended set of complementary dispersal statistics, including diffusion coefficients (Pybus *et al.* 2012, Trovão *et al.* 2015) and isolation-by-distance (IBD) signal metrics (Dellicour *et al.* 2024). While diffusion coefficients can be estimated to measure the diffusion velocity of lineages within the study area, IBD signal metrics aim to measure to what extent phylogenetic branches are spatially structured or the tendency of phylogenetically closely related tips to be sampled from geographically proximate locations (Dellicour *et al.* 2024). Together, these two metrics constitute a comprehensive framework that can e.g. be used to compare the dispersal capacities and patterns associated with different viruses spreading in various geographic areas and/or host and vector populations. Examples of lineage dispersal statistic metrics that can be estimated and visualized with “seraphim” are illustrated in Fig. 1B and C.

### 2.3 Investigating the impact of environmental factors on the lineage dispersal dynamics

The newly extended version of our R package now includes several analytical procedures, known as “landscape phylogeographic analyses” (Dellicour *et al.* 2018b), to investigate the impact of environmental factors on the dispersal dynamics of lineages. While the initial version of the package (Dellicour *et al.* 2016a) only allowed testing of associations between environmental factors and lineage dispersal velocities (Dellicour *et al.* 2017), this procedure has now been modified to focus on the diffusion coefficient instead of the dispersal velocity of lineages (Dellicour *et al.* 2025). This change, which is implemented in the “spreadFactors” function, was motivated by the fact that diffusion coefficients were found to be more robust to sampling intensity (i.e. the sampling size) than metrics based on lineage dispersal velocity (Bastide *et al.* 2024, Dellicour *et al.* 2024, Neher 2025). It has been demonstrated that, contrary to diffusion coefficient estimates, lineage dispersal velocity estimates tend to increase with the number of samples included in the continuous phylogeographic analysis, which can be explained by a mechanistic dependence of these metrics on the number of tip nodes in the trees (Dellicour *et al.* 2024, Neher 2025).

In addition to this post hoc procedure, “seraphim” now implements a prior-informed landscape phylogeographic approach that can be applied as an alternative method to investigate the impact of environmental factors on the diffusion velocity of lineages. In contrast to the post hoc approach, the new prior-informed procedure integrates environmental heterogeneity before conducting continuous phylogeographic inference. In short, the environmental factor under investigation is used to transform the space and we then test if the continuous phylogeographic reconstruction conducted in the transformed space leads to a more regular (i.e. more Brownian) diffusion velocity of lineages; this analysis can indicate if environmental factors are associated with a degree of heterogeneity in diffusion velocity (Dellicour *et al.* 2025). Such prior-informed landscape phylogeographic analyses can, for instance, be conducted through an environmental factor-based multidimensional scaling (MDS) transformation, using the “mdsTransformation” function implemented in “seraphim”. This prior-informed approach is conceptually different from the updated post hoc procedure detailed above, and there are advantages and disadvantages to both approaches, making them complementary. While the post hoc approach can use the flexibility of the RRW to relax the assumption of a constant dispersal velocity and to capture the impact of environmental factors, it is in general more suited for dispersal processes that remain correlated with geographic distance to some extent. Although this limitation does not apply to the prior-informed approach, the prior-informed method carries a higher computational burden, because in order to test each environmental raster it is necessary to conduct a distinct continuous phylogeographic analysis, which can become very time-consuming (Dellicour *et al.* 2025).

In addition to investigating the impact of environmental factors on the diffusion velocity of lineages, the “spreadFactors” function can be used to also test for associations between such environmental factors and the dispersal locations of lineages (Dellicour *et al.* 2019, 2020), i.e. to explore if inferred lineages have a tendency to preferentially circulate, or avoid circulating, in specific environmental conditions. Because it is directly based on the environmental values extracted at the tree node positions, and given the fact that half of those are sampling locations associated with tip nodes, this landscape phylogeographic approach is intrinsically related to and impacted by the sampling pattern. The results from this approach should therefore be interpreted with caution and in the light of the sampling effort. Finally, the “spreadFactors” function now also allows users to conduct isolation-by-resistance (IBR) analyses (Dellicour *et al.* 2025). These analyses can be performed to test to what extent environmental factors might be associated with a deviation from an IBD pattern.

### 2.4 Spatially explicit phylogeographic simulators

The “seraphim” package now includes four phylogeographic simulators implemented in distinct functions: (i) the function “simulatorRRW1” to conduct simulations of a RRW diffusion process along time-scaled phylogenies, which was used to investigate the impact of barriers on the dispersal frequency of lineages (Dellicour *et al.* 2018a; Klitting *et al.* 2022); (ii) the function “simulatorRRW2” to conduct simulations based on a birth-death process and a Brownian random walk (BRW) or a RRW diffusion process, which was used to assess the robustness of dispersal metrics estimated from continuous phylogeographic reconstructions (Dellicour *et al.* 2024); (iii) the function “simulatorRRW3” to conduct simulations of a RRW diffusion process with a dispersal velocity impacted by an environmental raster, which was used to evaluate the statistical performance of landscape phylogeographic approaches (Dellicour *et al.* 2025); and (iv) the function “treesRandomisations” to conduct tree branch randomization on an environmental raster according to various randomization procedures, with the possibility of an impact of the environmental values on the repulsion or attraction of lineages when randomizing the tree branches within the study area. The latter function implements the tree branch randomization procedure (Dellicour *et al.* 2016b) used by the “spreadFactors” function to generate a null dispersal model for statistical estimation in the different landscape phylogeographic approaches.

## 3 Example of data analysis and visualization

Figure 1 illustrates the kind of visualizations that can be generated using “seraphim”. These visualizations are based on a continuous phylogeographic analysis of the yellow fever virus (YFV) outbreak that started around 2015 in southeastern Brazil (Hill *et al.* 2022). The phylogeographic inference was based on an alignment of 466 complete YFV genomes sequenced from samples collected exclusively from non-human primates or from mosquitoes (i.e. excluding available human samples), thus focusing on the sylvatic transmission cycle of the virus. The data aimed to investigate the environmental factors impacting the dispersal dynamics of viral lineages within the sylvatic reservoir (Hill *et al.* 2022). In this figure, we display (i) a visualization of the continuous phylogeographic reconstruction made of three successive, cumulative snapshots, corresponding to three distinct points in time (August 2017 and 2018, as well as the most recent sampling date in April 2019; Fig. 1A), (ii) a visualization of the evolution through time of the maximal wavefront distance from the epidemic origin (Fig. 1B), and (iii) evaluation of the diffusion velocity of viral lineages through the estimation of the weighted diffusion coefficient (Fig. 1C).

## Supplementary Material

btag093_Supplementary_Data

## Data Availability

The R package “seraphim” is available at https://github.com/sdellicour/seraphim and continues to be developed. It is available along with example files and tutorials dedicated to the mapping of continuous phylogeographic reconstructions, the estimation of dispersal statistics, the use of the phylogeographic simulators implemented in the package, as well as the investigation of the impact of environmental factors on the dispersal dynamic of lineages (deviation from an isolation-by-distance pattern and impact on the diffusion velocity or dispersal location of lineages). The R package “seraphim” has several software dependencies and requires the prior installation of the following R packages: “ape”, “doMC” (only required for the MacOS version of “seraphim”), “fields”, “gdistance”, “HDInterval”, “ks”, “phytools”, “raster”, “RColorBrewer”, “R.utils”, and “vegan”. A commented version of the script used to generate the three panels of Fig. 1 is available in the following subdirectory of the GitHub repository: github.com/sdellicour/seraphim/tree/master/manuscript.

## References

[btag093-B1] Baele G , Dellicour S, Suchard MA et al Recent advances in computational phylodynamics. Curr Opin Virol 2018;31:24–32. 30248578 10.1016/j.coviro.2018.08.009

[btag093-B2] Baele G , Ji X, Hassler GW et al BEAST X for Bayesian phylogenetic, phylogeographic and phylodynamic inference. Nat Methods 2025;22:1653–6. 40624354 10.1038/s41592-025-02751-x PMC12328226

[btag093-B3] Bastide P , Rocu P, Wirtz J et al Modeling the velocity of evolving lineages and predicting dispersal patterns. Proc Natl Acad Sci USA 2024;121:e2411582121. 39546571 10.1073/pnas.2411582121 PMC11588136

[btag093-B4] Bouckaert R , Vaughan TG, Barido-Sottani J et al BEAST 2.5: an advanced software platform for Bayesian evolutionary analysis. PLOS Comput Biol 2019;15:e1006650. 30958812 10.1371/journal.pcbi.1006650 PMC6472827

[btag093-B5] De Maio N , Wu C-H, O’Reilly KM et al New routes to phylogeography: a Bayesian structured coalescent approximation. PLoS Genet 2015;11:e1005421. 26267488 10.1371/journal.pgen.1005421 PMC4534465

[btag093-B6] Dellicour S , Baele G, Dudas G et al Phylodynamic assessment of intervention strategies for the West African Ebola virus outbreak. Nat Commun 2018a;9:2222. 29884821 10.1038/s41467-018-03763-2 PMC5993714

[btag093-B7] Dellicour S , Bastide P, Rocu P et al How fast are viruses spreading in the wild? PLoS Biol 2024;22:e3002914. 39625970 10.1371/journal.pbio.3002914 PMC11614233

[btag093-B8] Dellicour S , Gámbaro F, Jacquot M et al Comparative performance of viral landscape phylogeography approaches. Proc Natl Acad Sci USA 2025;122:e2506743122. 40569388 10.1073/pnas.2506743122 PMC12232613

[btag093-B9] Dellicour S , Gill MS, Faria NR et al Relax, keep walking—a practical guide to continuous phylogeographic inference with BEAST. Mol Biol Evol 2021;38:3486–93. 33528560 10.1093/molbev/msab031 PMC8321535

[btag093-B10] Dellicour S , Lequime S, Vrancken B et al Epidemiological hypothesis testing using a phylogeographic and phylodynamic framework. Nat Commun 2020;11:5620. 33159066 10.1038/s41467-020-19122-z PMC7648063

[btag093-B11] Dellicour S , Rose R, Faria NR et al SERAPHIM: studying environmental rasters and phylogenetically informed movements. Bioinformatics 2016a;32:3204–6. 27334476 10.1093/bioinformatics/btw384

[btag093-B12] Dellicour S , Rose R, Faria NR et al Using viral gene sequences to compare and explain the heterogeneous spatial dynamics of virus epidemics. Mol Biol Evol 2017;34:2563–71. 28651357 10.1093/molbev/msx176

[btag093-B13] Dellicour S , Rose R, Pybus OG. Explaining the geographic spread of emerging epidemics: a framework for comparing viral phylogenies and environmental landscape data. BMC Bioinform 2016b;17:1–12. 10.1186/s12859-016-0924-x PMC4750353 26864798

[btag093-B14] Dellicour S , Troupin C, Jahanbakhsh F et al Using phylogeographic approaches to analyse the dispersal history, velocity, and direction of viral lineages—application to rabies virus spread in Iran. Mol Ecol 2019;28:4335–50. 31535448 10.1111/mec.15222

[btag093-B15] Dellicour S , Vrancken B, Trovão NS et al On the importance of negative controls in viral landscape phylogeography. Virus Evol2018b;4:vey023. 30151241 10.1093/ve/vey023 PMC6101606

[btag093-B16] Drummond AJ , Suchard MA, Xie D et al Bayesian phylogenetics with BEAUti and the BEAST 1.7. Mol Biol Evol 2012;29:1969–73. 22367748 10.1093/molbev/mss075 PMC3408070

[btag093-B17] Dudas G , Carvalho LM, Bedford T et al Virus genomes reveal factors that spread and sustained the Ebola epidemic. Nature 2017;544:309–15. 28405027 10.1038/nature22040 PMC5712493

[btag093-B18] Faria NR , Kraemer MUG, Hill SC et al Genomic and epidemiological monitoring of yellow fever virus transmission potential. Science 2018;361:894–9. 30139911 10.1126/science.aat7115 PMC6874500

[btag093-B19] Fisher AA , Ji X, Zhang Z et al Relaxed random walks at scale. Syst Biol 2021;70:258–67. 32687171 10.1093/sysbio/syaa056 PMC7875444

[btag093-B20] Guindon S , De Maio N. Accounting for spatial sampling patterns in Bayesian phylogeography. Proc Natl Acad Sci USA 2021;118:e2105273118. 34930835 10.1073/pnas.2105273118 PMC8719894

[btag093-B21] Hill SC , Dellicour S, Claro IM et al Climate and land-use shape the spread of zoonotic yellow fever virus. medRxiv 2022, preprint: not peer reviewed.

[btag093-B22] Kalkauskas A , Perron U, Sun Y et al Sampling bias and model choice in continuous phylogeography: getting lost on a random walk. PLoS Comput Biol 2021;17:e1008561. 33406072 10.1371/journal.pcbi.1008561 PMC7815209

[btag093-B23] Klitting R , Kafetzopoulou LE, Thiery W et al Predicting the evolution of the Lassa virus endemic area and population at risk over the next decades. Nat Commun 2022;13:5596. 36167835 10.1038/s41467-022-33112-3 PMC9515147

[btag093-B24] Kraemer MUG , Hill V, Ruis C et al; COVID-19 Genomics UK (COG-UK) Consortium. Spatiotemporal invasion dynamics of SARS-CoV-2 lineage B.1.1.7 emergence. Science (1979) 2021;373:889–95. 10.1126/science.abj0113 PMC9269003 34301854

[btag093-B25] Layan M , Müller NF, Dellicour S et al Impact and mitigation of sampling bias to determine viral spread: evaluating discrete phylogeography through CTMC modeling and structured coalescent model approximations. Virus Evol 2023;9:vead010. 36860641 10.1093/ve/vead010 PMC9969415

[btag093-B26] Lemey P , Rambaut A, Bedford T et al Unifying viral genetics and human transportation data to predict the global transmission dynamics of human influenza H3N2. PLoS Path 2014;10:e1003932. 10.1371/journal.ppat.1003932 PMC3930559 24586153

[btag093-B27] Lemey P , Rambaut A, Drummond AJ et al Bayesian phylogeography finds its roots. PLoS Comput Biol 2009;5:e1000520. 19779555 10.1371/journal.pcbi.1000520 PMC2740835

[btag093-B28] Lemey P , Rambaut A, Welch JJ et al Phylogeography takes a relaxed random walk in continuous space and time. Mol Biol Evol 2010;27:1877–85. 20203288 10.1093/molbev/msq067 PMC2915639

[btag093-B29] Müller NF , Bouckaert RR, Wu C-H et al MASCOT-skyline integrates population and migration dynamics to enhance phylogeographic reconstructions. PLoS Comput Biol 2025;21:e1013421. 41004543 10.1371/journal.pcbi.1013421 PMC12500135

[btag093-B30] Müller NF , Rasmussen D, Stadler T. MASCOT: parameter and state inference under the marginal structured coalescent approximation. Bioinformatics 2018;34:3843–8. 29790921 10.1093/bioinformatics/bty406 PMC6223361

[btag093-B31] Neher RA. Lost in the woods: shifting habitats can lead phylogeography astray. Virus Evol 2025;11:veaf040. 40584259 10.1093/ve/veaf040 PMC12202211

[btag093-B32] Pybus OG , Suchard MA, Lemey P et al Unifying the spatial epidemiology and molecular evolution of emerging epidemics. Proc Natl Acad Sci U S A 2012;109:15066–71. 22927414 10.1073/pnas.1206598109 PMC3443149

[btag093-B33] Trovão NS , Suchard MA, Baele G et al Bayesian inference reveals host-specific contributions to the epidemic expansion of Influenza A H5N1. Mol Biol Evol 2015;32:3264–75. 26341298 10.1093/molbev/msv185 PMC4831561

